# The genome sequence of the blonde ray, *Raja brachyura *Lafont, 1871

**DOI:** 10.12688/wellcomeopenres.22825.1

**Published:** 2024-08-08

**Authors:** Patrick Adkins, Rachel Brittain, Kesella Scott-Somme, Marine Biological Association Genome Acquisition Lab, Darwin Tree of Life Barcoding collective, Wellcome Sanger Institute Tree of Life Management, Samples and Laboratory team, Wellcome Sanger Institute Scientific Operations: Sequencing Operations, Wellcome Sanger Institute Tree of Life Core Informatics team, Tree of Life Core Informatics collective, Darwin Tree of Life Consortium

**Affiliations:** 1 The Marine Biological Association, Plymouth, England, UK

**Keywords:** Raja brachyura, blonde ray, genome sequence, chromosomal, Rajiformes

## Abstract

We present a genome assembly from an individual female *Raja brachyura* (Blonde Ray; Chordata; Chondrichthyes; Rajiformes; Rajidae). The genome sequence spans 2,700.50 megabases. Most of the assembly is scaffolded into 49 chromosomal pseudomolecules, including the X sex chromosome. The mitochondrial genome has also been assembled and is 17.12 kilobases in length. Gene annotation of this assembly on Ensembl identified 24,252 protein-coding genes.

## Species taxonomy

Eukaryota; Opisthokonta; Metazoa; Eumetazoa; Bilateria; Deuterostomia; Chordata; Craniata; Vertebrata; Gnathostomata; Chondrichthyes; Elasmobranchii; Batoidea; Rajiformes; Rajidae; *Raja*; *Raja brachyura,* Lafont, 1871 (NCBI:txid223862).

## Background

The Blonde Ray ( *Raja brachyura,* Lafont, 1871), is a benthic species of elasmobranch found in coastal waters around the north-east Atlantic. In UK waters it is more commonly found along the west and south coasts and typically shows a preference for sandy bottoms ( Serena, 2005). It is light brown with creamy-white patches and numerous dark brown/black spots which reach the edges of the of the wing and onto the tail ( Wheeler & Stebbing, 1978). It can reach sizes of 120cm from nose to tail and weigh up to 18 kg ( Stehmann *et al.*, 1984). The Blonde Ray has a varied diet of crustaceans, annelids and fish, with larger, more active crustaceans and fish becoming a more important component of the diet as the fish grows in size ( Ellis *et al.*, 1996; Holden & Tucker, 1974). The Blonde Ray mature between ages 4 and 6, and at around 80 cm in length (typically later and larger in females) ( Gallagher *et al.*, 2004). As with other Skates, Blond Rays are oviparous typically laying around 30 to 40 eggs in a season ( Holden *et al.*, 1971; Porcu *et al.*, 2015).

*Raja brachyura* is a commercially important species throughout its range ( Thys *et al.*, 2022). Its low fecundity, coupled with sexual maturity not being reached until the ray is large and several years old, makes the Blonde Ray vulnerable to fisheries pressure (both targeted and bycatch) ( Silva *et al.*, 2012; Thys *et al.*, 2022, Thys *et al.*, 2023). As such it is currently listed as near threatened with a decreasing population trend by the IUCN Redlist ( McCully *et al.*, 2015). Here we present the first chromosomally complete genome for this species and genus.

## Genome sequence report

**Figure 1.**
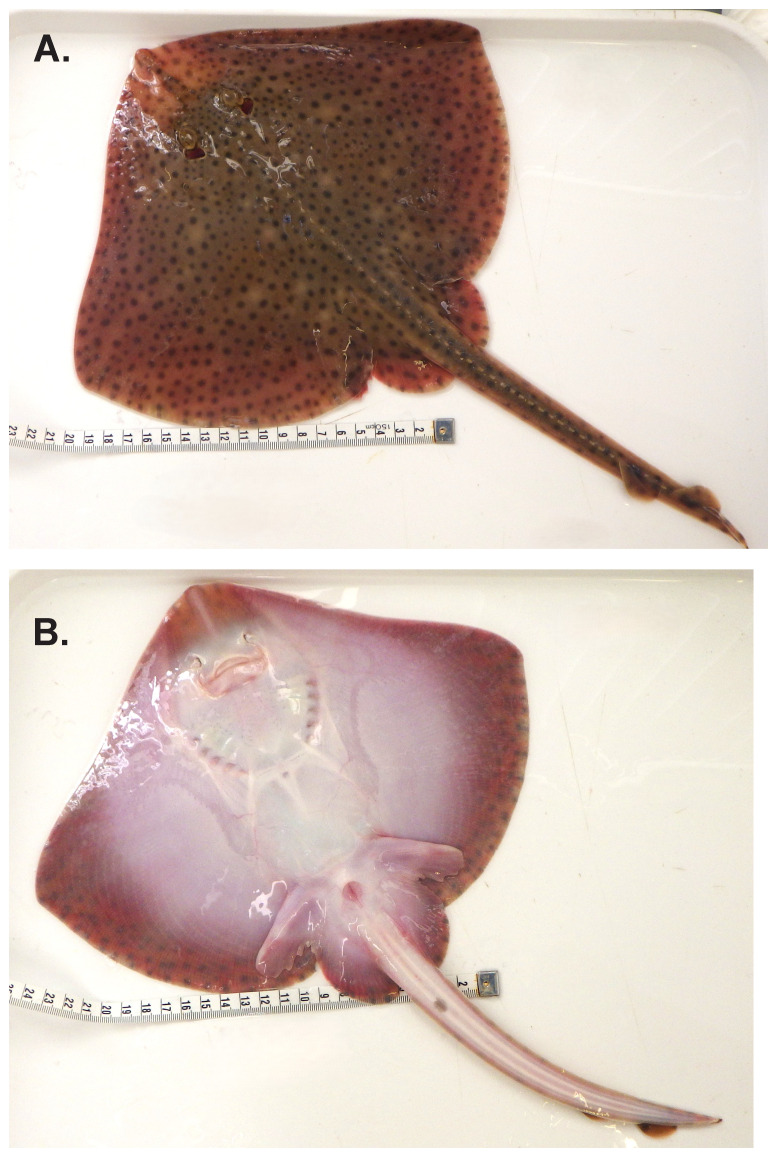
Photograph of the *Raja brachyura* (sRajBra1) specimen used for genome sequencing: **A**. Dorsal view, **B**. Ventral view.

The genome of an adult female *Raja brachyura* ( Figure 1) was sequenced using Pacific Biosciences single-molecule HiFi long reads, generating a total of 24.00 Gb (gigabases) from 1.92 million reads, providing approximately 33-fold coverage. Primary assembly contigs were scaffolded with chromosome conformation Hi-C data, which produced 630.99 Gbp from 4,178.75 million reads, yielding an approximate coverage of 234-fold. Specimen and sequencing information is summarised in Table 1.

**Table 1.** Specimen and sequencing data for *Raja brachyura*.

Project information			
**Study title**	Raja brachyura (blonde ray)		
**Umbrella BioProject**	PRJEB61690		
**Species**	*Raja brachyura*		
**BioSample**	SAMEA110450105		
**NCBI taxonomy ID**	223862		
Specimen information			
**Technology**	**ToLID**	**BioSample accession**	**Organism part**
**PacBio long read sequencing**	sRajBra1	SAMEA110450975	gill
**Hi-C sequencing**	sRajBra1	SAMEA110450974	gill
**RNA sequencing**	sRajBra1	SAMEA110450975	gill
Sequencing information			
**Platform**	**Run accession**	**Read count**	**Base count** **(Gb)**
**Hi-C Illumina NovaSeq 6000**	ERR11439618	4.18e+09	630.99
**PacBio Sequel IIe**	ERR11279096	1.70e+06	21.17
**PacBio Sequel IIe**	ERR11279097	1.69e+06	20.64
**PacBio Sequel IIe**	ERR11279098	1.94e+06	23.23
**PacBio Sequel IIe**	ERR11279099	1.92e+06	24.0
**RNA Illumina NovaSeq 6000**	ERR11837490	5.61e+07	8.47

**Figure 2.**
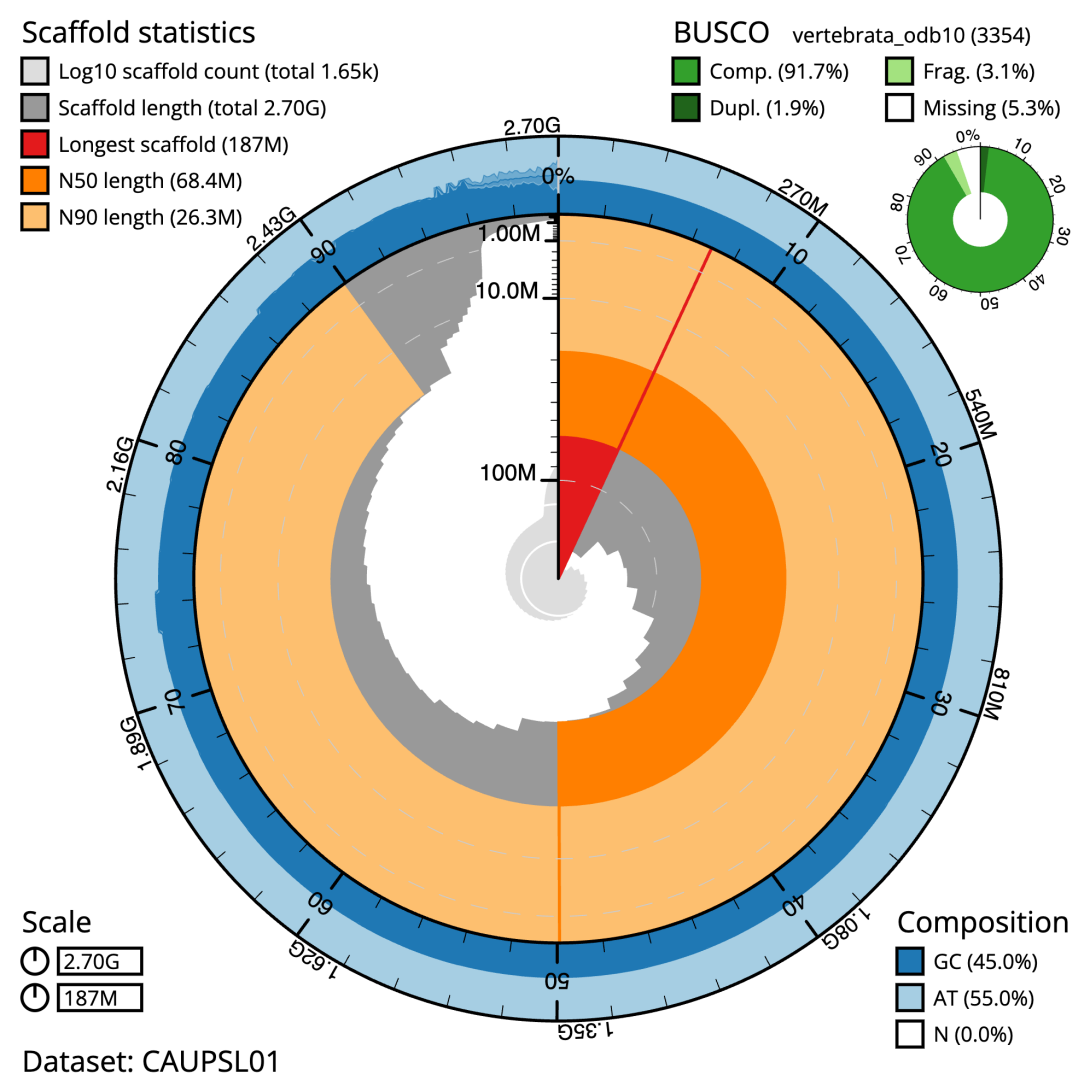
Genome assembly of *Raja brachyura*, sRajBra1.1: metrics. The BlobToolKit snail plot shows N50 metrics and BUSCO gene completeness. The main plot is divided into 1,000 size-ordered bins around the circumference with each bin representing 0.1% of the 2,700,467,673 bp assembly. The distribution of scaffold lengths is shown in dark grey with the plot radius scaled to the longest scaffold present in the assembly (187,439,314 bp, shown in red). Orange and pale-orange arcs show the N50 and N90 scaffold lengths (68,366,687 and 26,303,439 bp), respectively. The pale grey spiral shows the cumulative scaffold count on a log scale with white scale lines showing successive orders of magnitude. The blue and pale-blue area around the outside of the plot shows the distribution of GC, AT and N percentages in the same bins as the inner plot. A summary of complete, fragmented, duplicated and missing BUSCO genes in the vertebrata_odb10 set is shown in the top right. An interactive version of this figure is available at https://blobtoolkit.genomehubs.org/view/Raja%20brachyura/dataset/CAUPSL01/snail.

**Figure 3.**
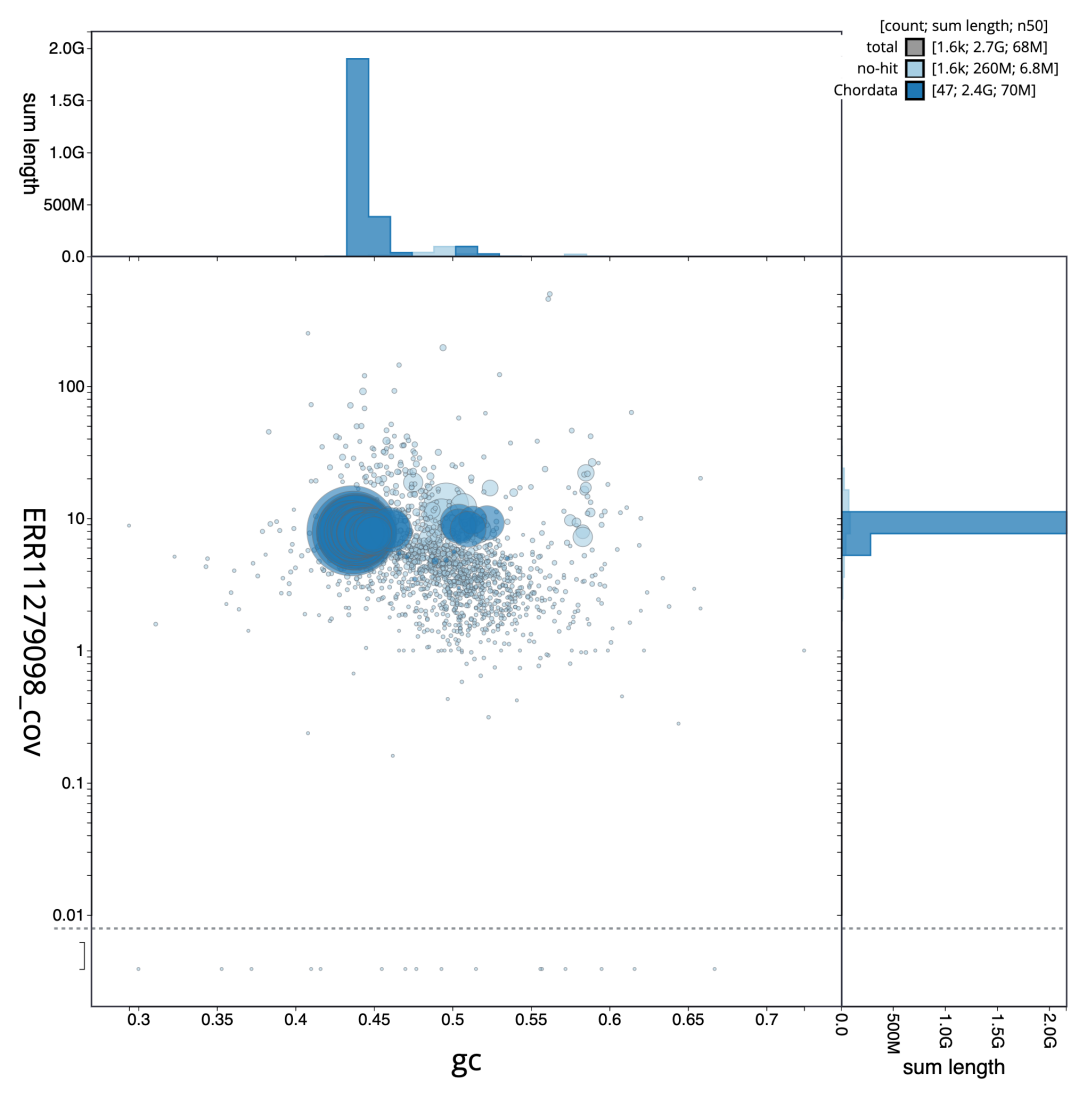
Genome assembly of *Raja brachyura*, sRajBra1.1: BlobToolKit GC-coverage plot. Sequences are coloured by phylum. Circles are sized in proportion to sequence length. Histograms show the distribution of sequence length sum along each axis. An interactive version of this figure is available at https://blobtoolkit.genomehubs.org/view/Raja%20brachyura/dataset/CAUPSL01/blob.

**Figure 4.**
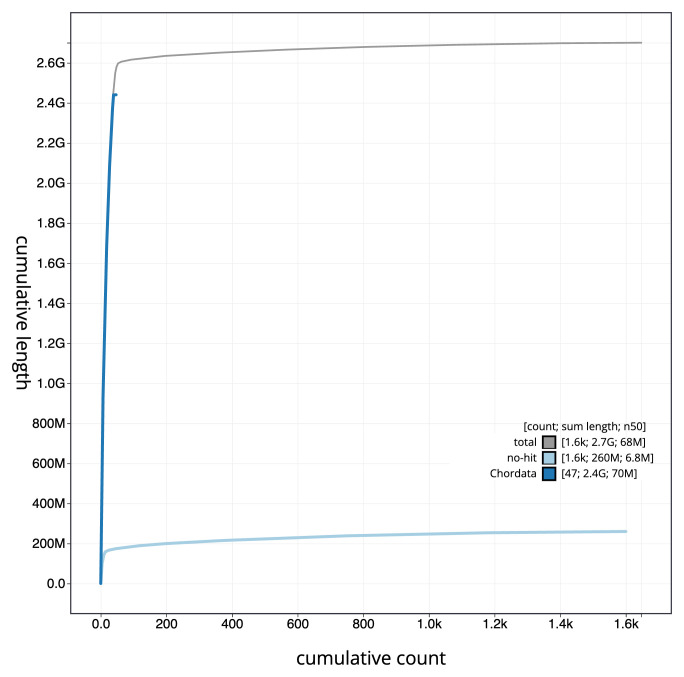
Genome assembly of *Raja brachyura* sRajBra1.1: BlobToolKit cumulative sequence plot. The grey line shows cumulative length for all sequences. Coloured lines show cumulative lengths of sequences assigned to each phylum using the buscogenes taxrule. An interactive version of this figure is available at https://blobtoolkit.genomehubs.org/view/Raja%20brachyura/dataset/CAUPSL01/cumulative.

**Figure 5.**
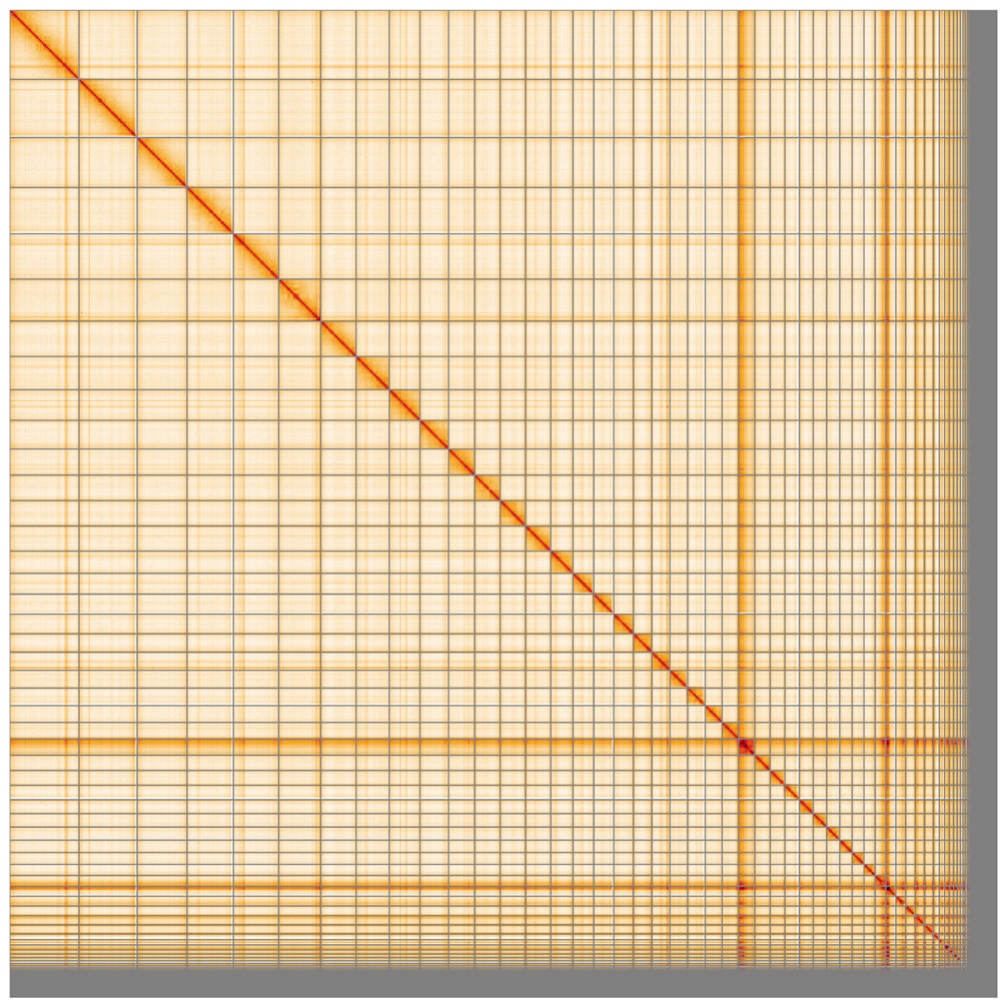
Genome assembly of *Raja brachyura* sRajBra1.1: Hi-C contact map of the sRajBra1.1 assembly, visualised using HiGlass. Chromosomes are shown in order of size from left to right and top to bottom. An interactive version of this figure may be viewed at https://genome-note-higlass.tol.sanger.ac.uk/l/?d=DS9CH9xQRVC81LrwHEfXaQ.

Manual assembly curation corrected 119 missing joins or mis-joins and one haplotypic duplications, reducing the scaffold number by 5.24%, and increasing the scaffold N50 by 0.57%. The final assembly has a total length of 2,700.50 Mb in 1,646 sequence scaffolds with a scaffold N50 of 68.4 Mb ( Table 2). The total count of gaps in the scaffolds is 2,269. The snail plot in Figure 2 provides a summary of the assembly statistics, while the distribution of assembly scaffolds on GC proportion and coverage is shown in Figure 3. The cumulative assembly plot in Figure 4 shows curves for subsets of scaffolds assigned to different phyla. Most (96.03%) of the assembly sequence was assigned to 49 chromosomal-level scaffolds, representing 48 autosomes and the X sex chromosome. Chromosome-scale scaffolds confirmed by the Hi-C data are named in order of size ( Figure 5; Table 3). The X chromosome was identified based on synteny with *Carcharodon carcharias* (GCA_017639515.1) and *Hypanus sabinus* (GCA_030144855.1). While not fully phased, the assembly deposited is of one haplotype. Contigs corresponding to the second haplotype have also been deposited. The mitochondrial genome was also assembled and can be found as a contig within the multifasta file of the genome submission.

**Table 2.** Genome assembly data for *Raja brachyura*, sRajBra1.1.

Genome assembly		
Assembly name	sRajBra1.1	
Assembly accession	GCA_963514005.1	
*Accession of alternate haplotype*	*GCA_963513955.1*	
Span (Mb)	2,700.50	
Number of contigs	3,916	
Contig N50 length (Mb)	2.0	
Number of scaffolds	1,646	
Scaffold N50 length (Mb)	68.4	
Longest scaffold (Mb)	187.44	
Assembly metrics *		*Benchmark*
Consensus quality (QV)	57.8	*≥ 50*
*k*-mer completeness	99.99%	*≥ 95%*
BUSCO **	C:91.7%[S:89.8%,D:1.9%],F:3.1%, M:5.2%,n:3,354	*C ≥ 95%*
Percentage of assembly mapped to chromosomes	96.03%	*≥ 95%*
Sex chromosomes	X	*localised homologous pairs*
Organelles	Mitochondrial genome: 17.12 kb	*complete single alleles*
Genome annotation of assembly GCA_963514005.1 at Ensembl		
Number of protein-coding genes	24,252	
Number of non-coding genes	2,564	
Number of gene transcripts	61,067	

* Assembly metric benchmarks are adapted from column VGP-2020 of “Table 1: Proposed standards and metrics for defining genome assembly quality” from Rhie *et al.* (2021). ** BUSCO scores based on the vertebrata_odb10 BUSCO set using version 5.4.3. C = complete [S = single copy, D = duplicated], F = fragmented, M = missing, n = number of orthologues in comparison. A full set of BUSCO scores is available at https://blobtoolkit.genomehubs.org/view/Raja%20brachyura/dataset/CAUPSL01/busco.

**Table 3.** Chromosomal pseudomolecules in the genome assembly of *Raja brachyura*, sRajBra1.

INSDC accession	Name	Length (Mb)	GC%
OY740781.1	1	187.44	43.5
OY740782.1	2	156.67	43.5
OY740783.1	3	135.09	44.0
OY740784.1	4	124.79	43.5
OY740785.1	5	122.43	44.0
OY740786.1	6	113.6	44.0
OY740787.1	7	95.33	43.5
OY740788.1	8	89.42	44.0
OY740789.1	9	82.82	44.0
OY740790.1	10	77.23	43.5
OY740791.1	11	70.46	44.0
OY740792.1	12	69.07	44.0
OY740793.1	13	68.37	44.0
OY740794.1	14	67.98	44.0
OY740795.1	15	59.78	44.5
OY740796.1	16	56.13	44.0
OY740797.1	17	53.96	44.0
OY740798.1	18	53.24	44.5
OY740799.1	19	49.69	44.5
OY740800.1	20	48.88	44.0
OY740801.1	21	48.0	45.0
OY740802.1	22	47.17	44.0
OY740803.1	23	45.74	44.0
OY740804.1	24	44.59	45.0
OY740805.1	25	43.39	49.5
OY740806.1	26	37.6	46.0
OY740807.1	27	41.72	45.0
OY740808.1	28	40.02	44.5
OY740809.1	29	38.76	45.0
OY740810.1	30	36.02	45.0
OY740811.1	31	35.16	46.0
OY740812.1	32	33.72	44.5
OY740813.1	33	31.87	44.5
OY740814.1	34	30.16	45.0
OY740815.1	35	29.07	50.5
OY740816.1	36	26.73	49.5
OY740818.1	37	26.3	45.0
OY740819.1	38	24.54	52.0
OY740820.1	39	24.5	50.5
OY740821.1	40	15.58	51.5
OY740822.1	41	14.02	51.5
OY740823.1	42	13.32	50.5
OY740824.1	43	11.17	50.0
OY740825.1	44	9.95	48.5
OY740826.1	45	8.55	46.5
OY740827.1	46	6.63	47.5
OY740828.1	47	4.4	48.5
OY740829.1	48	4.15	52.5
OY740817.1	X	26.66	51.0
OY740830.1	MT	0.02	41.0

The estimated Quality Value (QV) of the final assembly is 57.8 with *k*-mer completeness of 99.99%, and the assembly has a BUSCO v5.4.3 completeness of 91.7% (single = 89.8%, duplicated = 1.9%), using the vertebrata_odb10 reference set ( *n* = 3,354).

Metadata for specimens, BOLD barcode results, spectra estimates, sequencing runs, contaminants and pre-curation assembly statistics are given at https://tolqc.cog.sanger.ac.uk/darwin/sharks/Raja_brachyura/.

## Genome annotation report

The *Raja brachyura* genome assembly (GCA_963514005.1) was annotated at the European Bioinformatics Institute (EBI) on Ensembl Rapid Release. The resulting annotation includes 61,067 transcribed mRNAs from 24,252 protein-coding and 2,564 non-coding genes ( Table 2; https://rapid.ensembl.org/Raja_brachyura_GCA_963514005.1/Info/Index). The average transcript length is 68,391.85. There are 2.28 coding transcripts per gene and 13.47 exons per transcript.

## Methods

### Sample acquisition

A *Raja brachyura* specimen (specimen ID MBA-211013-003A, ToLID sRajBra1) was collected from Whitsand Bay, English Channel, UK (latitude 50.33, longitude –4.24) on 13th October 2021. The specimen was taken from its habitat of shell and sand using an otter trawl deployed from RV Sepia. The specimen was identified by Rachel Brittain (Marine Biological Association) based on gross morphology. The fish was first anaesthetised and then overdosed using Aquased (2-phenoxyethanol). Destruction of the brain was used as a secondary method to ensure the animal was deceased before tissue sampling took place as in accordance with Schedule 1 methodology under the home office licence. Samples taken from the animal were preserved on dry ice.

The initial identification was verified by an additional DNA barcoding process according to the framework developed by Twyford *et al*. (2024). A small sample was dissected from the specimen and stored in ethanol, while the remaining parts of the specimen were shipped on dry ice to the Wellcome Sanger Institute (WSI). The tissue was lysed, the COI marker region was amplified by PCR, and amplicons were sequenced and compared to the BOLD database, confirming the species identification ( Crowley *et al.*, 2023). Following whole genome sequence generation, the relevant DNA barcode region was also used alongside the initial barcoding data for sample tracking at the WSI ( Twyford *et al.*, 2024). The standard operating procedures for Darwin Tree of Life barcoding have been deposited on protocols.io ( Beasley *et al.*, 2023).

### Nucleic acid extraction

The workflow for high molecular weight (HMW) DNA extraction at the Wellcome Sanger Institute (WSI) Tree of Life Core Laboratory includes a sequence of core procedures: sample preparation; sample homogenisation, DNA extraction, fragmentation, and clean-up. In sample preparation, the sRajBra1 sample was weighed and dissected on dry ice ( Jay *et al.*, 2023). Gill tissue was homogenised using a PowerMasher II tissue disruptor ( Denton *et al.*, 2023a). HMW DNA was extracted using the Nanobind Blood extraction protocol ( Denton *et al.*, 2023b). DNA was sheared into an average fragment size of 12–20 kb in a Megaruptor 3 system ( Todorovic *et al.*, 2023). Sheared DNA was purified by solid-phase reversible immobilisation ( Strickland *et al.*, 2023): in brief, the method employs AMPure PB beads to eliminate shorter fragments and concentrate the DNA. The concentration of the sheared and purified DNA was assessed using a Nanodrop spectrophotometer and Qubit Fluorometer using the Qubit dsDNA High Sensitivity Assay kit. Fragment size distribution was evaluated by running the sample on the FemtoPulse system. 

RNA was extracted from gill tissue of sRajBra1 in the Tree of Life Laboratory at the WSI using the RNA Extraction: Automated MagMax™ *mir*Vana protocol ( do Amaral *et al.*, 2023). The RNA concentration was assessed using a Nanodrop spectrophotometer and a Qubit Fluorometer using the Qubit RNA Broad-Range Assay kit. Analysis of the integrity of the RNA was done using the Agilent RNA 6000 Pico Kit and Eukaryotic Total RNA assay.

Protocols developed by the WSI Tree of Life laboratory are publicly available on protocols.io ( Denton *et al.*, 2023c).

### Sequencing

Pacific Biosciences HiFi circular consensus DNA sequencing libraries were constructed according to the manufacturers’ instructions. Poly(A) RNA-Seq libraries were constructed using the NEB Ultra II RNA Library Prep kit. DNA and RNA sequencing was performed by the Scientific Operations core at the WSI on Pacific Biosciences Sequel IIe (HiFi) and Illumina NovaSeq 6000 (RNA-Seq) instruments. Hi-C data were also generated from gill tissue of sRajBra1 using the Arima-HiC v2 kit. The Hi-C sequencing was performed using paired-end sequencing with a read length of 150 bp on the Illumina NovaSeq 6000 instrument.

### Genome assembly, curation and evaluation

***Assembly***

The original assembly of HiFi reads was performed using Hifiasm ( Cheng *et al.*, 2021) with the --primary option. Haplotypic duplications were identified and removed with purge_dups ( Guan *et al.*, 2020). Hi-C reads are further mapped with bwa-mem2 ( Vasimuddin *et al.*, 2019) to the primary contigs, which are further scaffolded using the provided Hi-C data ( Rao *et al.*, 2014) in YaHS ( Zhou *et al.*, 2023) using the --break option. Scaffolded assemblies are evaluated using Gfastats ( Formenti *et al.*, 2022), BUSCO ( Manni *et al.*, 2021) and MERQURY.FK ( Rhie *et al.*, 2020).

The mitochondrial genome was assembled using MitoHiFi ( Uliano-Silva *et al.*, 2023), which runs MitoFinder ( Allio *et al.*, 2020) and uses these annotations to select the final mitochondrial contig and to ensure the general quality of the sequence.

***Assembly curation***

The assembly was decontaminated using the Assembly Screen for Cobionts and Contaminants (ASCC) pipeline (article in preparation). Flat files and maps used in curation were generated in TreeVal ( Pointon *et al.*, 2023). Manual curation was primarily conducted using PretextView ( Harry, 2022), with additional insights provided by JBrowse2 ( Diesh *et al.*, 2023) and HiGlass ( Kerpedjiev *et al.*, 2018). Scaffolds were visually inspected and corrected as described by Howe *et al*. (2021). Any identified contamination, missed joins, and mis-joins were corrected, and duplicate sequences were tagged and removed. Sex chromosomes were identified by synteny. The entire process is documented at https://gitlab.com/wtsi-grit/rapid-curation (article in preparation).

***Evaluation of the final assembly***

A Hi-C map for the final assembly was produced using bwa-mem2 ( Vasimuddin *et al.*, 2019) in the Cooler file format ( Abdennur & Mirny, 2020). To assess the assembly metrics, the *k*-mer completeness and QV consensus quality values were calculated in Merqury ( Rhie *et al.*, 2020). This work was done using Nextflow ( Di Tommaso *et al.*, 2017) DSL2 pipelines “sanger-tol/readmapping” ( Surana *et al.*, 2023a) and “sanger-tol/genomenote” ( Surana *et al.*, 2023b). The genome was analysed within the BlobToolKit environment ( Challis *et al.*, 2020) and BUSCO scores ( Manni *et al.*, 2021; Simão *et al.*, 2015) were calculated.

The genome assembly and evaluation pipelines were developed using the nf-core tooling ( Ewels *et al.*, 2020), use MultiQC ( Ewels *et al.*, 2016), and make extensive use of the Conda package manager, the Bioconda initiative ( Grüning *et al.*, 2018), the Biocontainers infrastructure ( da Veiga Leprevost *et al.*, 2017), and the Docker ( Merkel, 2014) and Singularity ( Kurtzer *et al.*, 2017) containerisation solutions.

Table 4 contains a list of relevant software tool versions and sources.

**Table 4.** Software tools: versions and sources.

Software tool	Version	Source
BEDTools	2.30.0	https://github.com/arq5x/bedtools2
BLAST	2.14.0	ftp://ftp.ncbi.nlm.nih.gov/blast/executables/blast+/
BlobToolKit	4.3.7	https://github.com/blobtoolkit/blobtoolkit
BUSCO	5.4.3 and 5.5.0	https://gitlab.com/ezlab/busco
bwa-mem2	2.2.1	https://github.com/bwa-mem2/bwa-mem2
Cooler	0.8.11	https://github.com/open2c/cooler
DIAMOND	2.1.8	https://github.com/bbuchfink/diamond
fasta_windows	0.2.4	https://github.com/tolkit/fasta_windows
FastK	427104ea91c78c3b8b8b49f1a7d6bbeaa869ba1c	https://github.com/thegenemyers/FASTK
Gfastats	1.3.6	https://github.com/vgl-hub/gfastats
GoaT CLI	0.2.5	https://github.com/genomehubs/goat-cli
Hifiasm	0.16.1-r375	https://github.com/chhylp123/hifiasm
HiGlass	44086069ee7d4d3f6f3f0012569789ec138f42b84 aa44357826c0b6753eb28de	https://github.com/higlass/higlass
Merqury.FK	d00d98157618f4e8d1a9190026b19b471055b22e	https://github.com/thegenemyers/MERQURY.FK
MitoHiFi	3	https://github.com/marcelauliano/MitoHiFi
MultiQC	1.14, 1.17, and 1.18	https://github.com/MultiQC/MultiQC
NCBI Datasets	15.12.0	https://github.com/ncbi/datasets
Nextflow	23.04.0-5857	https://github.com/nextflow-io/nextflow
PretextView	0.2	https://github.com/sanger-tol/PretextView
purge_dups	1.2.5	https://github.com/dfguan/purge_dups
samtools	1.16.1, 1.17, and 1.18	https://github.com/samtools/samtools
sanger-tol/ascc	-	https://github.com/sanger-tol/ascc
sanger-tol/genomenote	1.1.1	https://github.com/sanger-tol/genomenote
sanger-tol/readmapping	1.2.1	https://github.com/sanger-tol/redmapping
Seqtk	1.3	https://github.com/lh3/seqtk
Singularity	3.9.0	https://github.com/sylabs/singularity
TreeVal	1.0.0	https://github.com/sanger-tol/treeval
YaHS	1.2a.2	https://github.com/c-zhou/yahs

### Genome annotation

The Ensembl Genebuild annotation system ( Aken *et al.*, 2016) was used to generate annotation for the *Raja brachyura* assembly (GCA_963514005.1) in Ensembl Rapid Release at the EBI. Annotation was created primarily through alignment of transcriptomic data to the genome, with gap filling via protein-to-genome alignments of a select set of proteins from UniProt ( UniProt Consortium, 2019).

### Wellcome Sanger Institute – Legal and Governance

The materials that have contributed to this genome note have been supplied by a Darwin Tree of Life Partner. The submission of materials by a Darwin Tree of Life Partner is subject to the **‘Darwin Tree of Life Project Sampling Code of Practice’**, which can be found in full on the Darwin Tree of Life website here. By agreeing with and signing up to the Sampling Code of Practice, the Darwin Tree of Life Partner agrees they will meet the legal and ethical requirements and standards set out within this document in respect of all samples acquired for, and supplied to, the Darwin Tree of Life Project. 

Further, the Wellcome Sanger Institute employs a process whereby due diligence is carried out proportionate to the nature of the materials themselves, and the circumstances under which they have been/are to be collected and provided for use. The purpose of this is to address and mitigate any potential legal and/or ethical implications of receipt and use of the materials as part of the research project, and to ensure that in doing so we align with best practice wherever possible. The overarching areas of consideration are:

●   Ethical review of provenance and sourcing of the material

●   Legality of collection, transfer and use (national and international)

Each transfer of samples is further undertaken according to a Research Collaboration Agreement or Material Transfer Agreement entered into by the Darwin Tree of Life Partner, Genome Research Limited (operating as the Wellcome Sanger Institute), and in some circumstances other Darwin Tree of Life collaborators.

## Data Availability

European Nucleotide Archive: *Raja brachyura* (blonde ray). Accession number PRJEB61690; https://identifiers.org/ena.embl/PRJEB61690 ( Wellcome Sanger Institute, 2023). The genome sequence is released openly for reuse. The *Raja brachyura* genome sequencing initiative is part of the Darwin Tree of Life (DToL) project. All raw sequence data and the assembly have been deposited in INSDC databases. Raw data and assembly accession identifiers are reported in Table 1 and Table 2.

## Author information

Members of the Marine Biological Association Genome Acquisition Lab are listed here: https://doi.org/10.5281/zenodo.8382513.

Members of the Darwin Tree of Life Barcoding collective are listed here: https://doi.org/10.5281/zenodo.4893703.

Members of the Wellcome Sanger Institute Tree of Life Management, Samples and Laboratory team are listed here: https://doi.org/10.5281/zenodo.10066175.

Members of Wellcome Sanger Institute Scientific Operations: Sequencing Operations are listed here: https://doi.org/10.5281/zenodo.10043364.

Members of the Wellcome Sanger Institute Tree of Life Core Informatics team are listed here: https://doi.org/10.5281/zenodo.10066637.

Members of the Tree of Life Core Informatics collective are listed here: https://doi.org/10.5281/zenodo.5013541.

Members of the Darwin Tree of Life Consortium are listed here: https://doi.org/10.5281/zenodo.4783558.
