# Supplementary material for: The genome sequence of the blonde ray, Raja brachyura Lafont, 1871
Source: Wellcome Open Res. 2024 Aug 8;9:436. [Version 1] doi: 10.12688/wellcomeopenres.22825.1 (PMC11358690; doi:10.12688/wellcomeopenres.22825.1)
Supplement: Peer review history

# Reviewer report: Reviewer response for version 1

**DOI:** 10.21956/wellcomeopenres.25132.r93541

**Published:** 2024-09-11

**Authors:** Shawn M. Burgess

**Affiliations:** 1 National Human Genome Research Institute, Bethesda, USA

The authors have submitted a good quality assembly of the blond ray, *Raja brachyura*. The assembly was done with PacBio HiFi and HiC using the Hifiasm assembler which is a standard approach at this point for good draft assemblies. I do not have any major concerns with the approach or results. It would be interesting to see a comparison between the primary haplotype and the secondary contigs. What is the snv rate between the two chromosomes? How much genetic diversity is stored in the shrinking populations of blond rays? 

 These are not essential points, but are potentially of interest.

Are sufficient details of methods and materials provided to allow replication by others?

Yes

Is the rationale for creating the dataset(s) clearly described?

Yes

Are the datasets clearly presented in a useable and accessible format?

Yes

Are the protocols appropriate and is the work technically sound?

Yes

Reviewer Expertise:

Zebrafish genomics, fish genome assembly, tissue regeneration.

I confirm that I have read this submission and believe that I have an appropriate level of expertise to confirm that it is of an acceptable scientific standard.

# Reviewer report: Reviewer response for version 1

**DOI:** 10.21956/wellcomeopenres.25132.r93546

**Published:** 2024-08-28

**Authors:** Stephan Koblmüller

**Affiliations:** 1 University of Graz, Graz, Austria

Adkins et al. present the genome assembly of the blonde ray, *Raja brachyura*. The data are clear and the approaches are well described and I have no objections to any of the data presented. As far as I can tell, the datasets are deposited in publicly accessible repositories.

 The background section gives a concise, but nonetheless pretty comprehensive introduction to the study species. Though it is clear to me that providing a high-quality reference genome for this species will serve as basis for numerous other studies, the rationale of why actually sequencing a high-quality reference genome for this species is missing in the background section. In my opinion, one to two sentences on the rationale are required.

 In addition, I think, one sentence needs to be rephrased: The Blonde Ray mature between ages 4 and 6, and at around 80 cm in length (typically later and larger in females) (Gallagher et al. 2004) Here “mature” should be replaced by “matures” and I guess with “between ages 4 and 6” you mean between 4 and 6 years, right? I suggest you rephrase this so that it’s clear for the reader what you mean.

Are sufficient details of methods and materials provided to allow replication by others?

Yes

Is the rationale for creating the dataset(s) clearly described?

Partly

Are the datasets clearly presented in a useable and accessible format?

Yes

Are the protocols appropriate and is the work technically sound?

Yes

Reviewer Expertise:

phylogenetics-/genomics, population genetics/genomics

I confirm that I have read this submission and believe that I have an appropriate level of expertise to confirm that it is of an acceptable scientific standard.

# Reviewer report: Reviewer response for version 1

**DOI:** 10.21956/wellcomeopenres.25132.r93550

**Published:** 2024-08-28

**Authors:** Jayan D.M Senevirathna

**Affiliations:** 1 Uva Wellassa University, Badulla, Uva Province, Sri Lanka

I accept this article for indexing in Open research.

**However, the followings are suggested to improve the article:**

- In keywords; *Raja brachyura should be italic.*
- In background; north-east Atlantic > north-east Atlantic Ocean.
- In Genome annotation report; Recheck the number of non-coding genes in the text and the given link.
- In methods; For HMW DNA extraction, why did you specifically select the gill tissue instead of a muscle...?

Are sufficient details of methods and materials provided to allow replication by others?

Yes

Is the rationale for creating the dataset(s) clearly described?

Yes

Are the datasets clearly presented in a useable and accessible format?

Yes

Are the protocols appropriate and is the work technically sound?

Yes

Reviewer Expertise:

Genetics and biotechnology

I confirm that I have read this submission and believe that I have an appropriate level of expertise to confirm that it is of an acceptable scientific standard.
